# The Well-Being of the German Adult Population Measured with the WHO-5 over Different Phases of the COVID-19 Pandemic: An Analysis within the COVID-19 Snapshot Monitoring Study (COSMO)

**DOI:** 10.3390/ijerph19063236

**Published:** 2022-03-09

**Authors:** Fang-Yi Tsai, Hannah Schillok, Michaela Coenen, Christina Merkel, Caroline Jung-Sievers, on behalf of the COSMO Study Group

**Affiliations:** 1 Chair of Public Health and Health Services Research, Institute for Medical Information Processing, Biometry, and Epidemiology—IBE, Elisabeth-Winterhalter-Weg 6, 81377 Munich, Germany; fangyi.tsai0809@gmail.com (F.-Y.T.); hannah.schillok@ibe.med.uni-muenchen.de (H.S.); coenen@ibe.med.uni-muenchen.de (M.C.); 2 Pettenkofer School of Public Health, Elisabeth-Winterhalter-Weg 6, 81377 Munich, Germany; 3 Federal Centre for Health Education (BZgA), Maar-Weg 149-161, 50825 Cologne, Germany; christina.merkel@bzga.de

**Keywords:** COVID-19, depression, mental health, WHO-5, psychological distress

## Abstract

The aim of this study is to evaluate factors associated with the subjective well-being (SWB) and suspected depression measured with WHO-5 among German adults during different phases of the COVID-19 pandemic. Survey data were analyzed from the COVID-19 Snapshot Monitoring (COSMO) study, which collected data from 972, 1013, and 973 participants in time point 1 (19–20 May 2020), time point 2 (15–16 September 2020), and time point 3 (21–22 December 2020), respectively. Descriptive analyses and logistic regression analyses to identify the factors associated with suspected depression (WHO-5 ≤ 50) were conducted. Data showed that the mean WHO-5 scores in three time points were 56.17, 57.27, and 53.93, respectively. The risk of suspected depression was increased by about 1.5 times for females, 2.5–3 times among 18–24 year-olds compared to ages above 65 years, 1.5 times for singles, 2 times for those with chronic illnesses, and 2–3 times for people living in poverty. The main study findings show that German adult SWB is lower than pre-pandemic reference values. Special focus should be placed on vulnerable groups, such as females, younger persons, and people living in poverty who are most prone to a reduction in SWB and therefore suspected depression.

## 1. Introduction

The coronavirus disease 2019 (COVID-19) pandemic presented great challenges to all aspects of the health system, resulting in global morbidity and mortality worldwide. Additionally, since the outbreak, people around the world experienced significant impacts on their well-being and mental health. Various measures, such as physical distancing and the suspension of services to intervene in the spread of the coronavirus, brought mental health implications to the public discussion [1]. Stressors, such as social isolation, vast human loss, fear of contagion, novel living situations, financial strain, and uncertainty of employment and shelter, may impact subjective well-being (SWB) and thus mental health in the medium and long term [1,2,3].

It is well known that stress affects the psychological and biological systems associated with anxiety and depression [4,5,6,7]. Stress might lead to an increase in inflammations, which contribute to depression pathogenesis [8]. Research comparing clinical mental health diagnosis and SWB scores suggest that a reduction in the SWB score may point to a suspected underlying depression, even though the SWB instruments do not qualify for clinical application [9]. To evaluate the measures against COVID-19, it is vital to comprehend the socioeconomic effects of the policies to manage the pandemic, which would inevitably have negative effects on mental health through increasing financial insecurity and unemployment rates [2]. However, even though stress is negatively correlated with SWB, this relationship was found to be completely mediated by the individual’s coping strategy [10]. This makes protective factors associated with SWB, as, e.g., the availability of personal resources, even more important, as those with sound strategies to cope with stress tend to experience less psychological distress.

While psychological support might in parts be provided to patients and healthcare workers during the COVID-19 pandemic, the public’s mental health requires significant attention as well [11]. The prevalence rate of psychological morbidities with respect to the event impact during the COVID-19 pandemic was 44% in the general population in China, Spain, Italy, Iran, the U.S., Turkey, Nepal, and Denmark, with stress (36%) being the most common problem, followed by poor sleep quality (34%), and psychological distress (26%), as reported by Krishnamoorthy et al. [12]. One study, including the data presented in this study, reported that a COVID-19 Snapshot Monitoring (COSMO) study revealed increased rates of depression, loneliness, and hopelessness in the German population during the COVID-19 pandemic, compared to the times before the pandemic [13]. This aligns with a recently published international meta-analysis focusing on the early COVID-19 pandemic phase, which confirmed increased symptoms of anxiety and depression in the general population [14]. Similar situations with higher psychological distress were also observed in previous outbreaks, such as Severe Acute Respiratory Syndrome (SARS), H1N1 influenza, Ebola virus and Middle East Respiratory Syndrome (MERS) [15,16]. Although most people are not expected to develop mental illnesses, it is still postulated that the majority of people would experience an emotional adjustment [17]. Thus, evidence from existing COVID-19 studies as well as past health crises indicate that this pandemic has a major impact on the SWB of the inhabitants. Since there has not been a comparable health crisis similar to the COVID-19 pandemic in Central Europe for a long time, the knowledge of how the general public can cope with it over a longer period of time, and the aspects that might influence their ability, is limited.

Our aim is therefore to add to this evidence by analyzing the factors associated with SWB and suspected depression in the adult German population during different phases of the pandemic, considering different risk or protective context factors measured in COSMO. The hypothesis is that the SWB of the general population changes over time and is dependent on: (1) the measures taken to contain the outbreak in different phases, such as contact restrictions, school closures, quarantine, and the suspension of services, and (2) different demographic and socioeconomic factors and health conditions.

## 2. Materials and Methods

### 2.1. Study Sample—The COSMO Study

The German COVID-19 Snapshot Monitoring (COSMO) study is a repeated cross-sectional survey study used to capture the broad psychosocial status of the German population during the pandemic. COSMO is a joint project by the University of Erfurt, the Robert Koch Institute (RKI), the Federal Centre for Health Education (BZgA), the Leibniz Institute for Psychology Information (ZPID), the Science Media Centre, the Bernhard Nocht Institute for Tropical Medicine, and the Yale Institute for Global Health [18]. The study undertook weekly or bi-weekly turns (called “waves”) in Germany, having commenced on 3 March 2020, using online questionnaires. For each wave, quota sampling was applied and was matched to the general German population in terms of age, gender, and federal states. To detect minor effects and increase the probability of congruence between the distribution of the demographics in the sample and the German population, a sample size of n = 1000 was chosen by the study team [18]. With a sensitivity power analysis for zero-order correlations (*p* = 0.05), a sample size of n = 1000 was supposed to be sufficient to detect the correlation coefficients of (at least) r = 0.08 with a sufficient power of 0.8 in each survey wave. The rationale for the study design, population selection, recruitment, and methods are further described in the COSMO study protocol. Participants were recruited via an external study sample provider according to ISO 26362. Before starting the survey, all participants were informed about the study and provided informed consent. They participated voluntarily and obtained remuneration. Ethical approval of the COSMO study was approved by the Institutional Review Board of University of Erfurt (#20200302/20200501).

Approximately n = 1000 people aged 18 to 74 years were questioned about their individual psychosocial situation, their knowledge about COVID-19, as well as their attitudes towards several institutions, authorities, and measures to contain the pandemic in each wave. We analyzed the subjective well-being (SWB) via WHO-5 and compared the data from three waves (time point 1 (wave 12 from 19–20 May 2020), time point 2 (wave 21 from 15–16 September 2020), and time point 3 (wave 31 from 21–22 December 2020)). The details of the study, including the study design, data acquisition, settings, and ethical standards are described elsewhere [18].

### 2.2. Evaluation Time Points

On 23 March 2020, the German Federal government introduced a strict lockdown to control the spread of the virus with a range of measures, including the enforcement of contact restrictions, social distancing requirements, and the closure of schools and non-essential businesses. On 20 April 2020, smaller shops were allowed to reopen respecting the rules of social distancing. On 6 May 2020, the government announced the further easing of containment measures for all shops and facilities. After 7 weeks of lockdown, the daily new COVID-19 cases reduced to less than 500. Since 26 May 2020, the federal and state governments eased restrictions on public gatherings for up to 10 people or 2 separate households, making it possible to meet in larger groups again. Since August, the number of new infections increased again, with more than 1000 new infections per day. Stricter measures and the “lockdown light” were introduced in 2 November 2020, whereby private gatherings were limited to a maximum of 5 persons from 2 households. Restaurants, facilities, and personal services providers were closed nationwide, although schools remained open. Since 16 December 2020, the lockdown was tightened by virtue of sustained high infection rates and rising death rates. This time, all non-essential shops, as well as schools and daycare centers were closed. The closures were scheduled until the end of January 2021 and extended to early March 2021 [19].

Our study used the data of the COSMO survey collected for time point 1 (19–20 May 2020), to analyze the SWB after the relaxation of the first strict lockdown; time point 2 (15–16 September 2020), to identify the SWB when people were required to maintain social distancing and hygiene measures; and time point 3 (21–22 December 2020), the time people experienced a second hard lockdown during the Christmas season. Based on the design of the COSMO study, each wave consists of approximately 1000 people that are matched to the general German population in terms of age, gender, and distribution across federal states. Following the described sampling strategy, general population characteristics in the three time points were comparable. However, minor invariances between the waves occurred as presented in Table 1.

### 2.3. Variables and Measures

#### 2.3.1. WHO-5

The subjective well-being (SWB) was assessed using WHO-5. WHO-5 is a generic, validated and widely used self-report scale, which may be applied as a screening tool for suspected depression. The psychometric properties, including internal consistency and test–retest reliability were reported in various settings and studies [20]. The WHO-5 is a validated scoring instrument with five items applied for the measurement of SWB over the previous fortnight (“felt cheerful and in good spirits”/“felt calm and relaxed”/“felt active and vigorous”/“woke up feeling fresh and rested”/“daily life filled with things that interest me”) [20]. Each item is to be answered on a 6-point scale ranging from 0 (“at no time”) to 5 (“all of the time”). The summed score of the 5 items leads to a raw score ranging from 0 (absence of SWB) to 25 (maximal SWB) and is multiplied by 4, mapping to a scale ranging from 0 to 100 (WHO-5 transformed). In the following, the transformed WHO-5 is refered to as WHO-5_T_.

According to Bech et al. [21], transformed scores between 0 and 25 represent poor SWB, scores between 26 and 50 represent fair SWB, scores between 51and 75 represent good SWB, and, lastly, scores between 76 and 100 represent very good SWB. A raw score below 13 indicates a poor SWB and is widely used as an indicator for screening for depression according to ICD-10 [22]. Similarly, the WHO-5_T_ is a recognized screening tool for suspected depression with a cut-off score of 50 or below with a sensitivity of 0.86 and a specificity of 0.81 [20]. Persons with a WHO-5 _T_ score of 50 or below are classified as persons “screened for depression” and persons with “suspected depression” (or being at risk of suspected depression, respectively).

#### 2.3.2. Demographic Variables

Demographic variables, such as gender (female, male); age (18–24, 25–34, 35–49, 50–64, and ≥65 years); education level (with or without A-levels (German university entrance qualification)); migration background awareness (“Are you aware of yourself or any of your parents being born abroad?”: yes, no, do not know); household language (German or other than German); relationship status (relationship or partnership, including marriage: yes, no); age of the participant’s children (multiple choices: 0–5 years old, 6–13 years old, and 14–17 years old); and single parents (only for respondents who have children: yes, no) were assessed (see also Table A8).

#### 2.3.3. Socioeconomic Variables

Socioeconomic variables, such as employment (yes, no); status as working in the health sector (yes, no); self-employed (yes, no); and household size (just me, 2 persons, 3 or more persons) were assessed. The number of inhabitants of a hometown was classified into 4 categories: ≤20,000 (small town); 20,001–100,000 (medium-sized town); 100,001–500,000 (city); and >500,000 (big city) inhabitants. Monthly household net income was classified according to the Federal Statistical Office of Germany, the threshold for the risk of poverty in 2019 was EUR 14,109 for a single household, which translates to roughly EUR 1175 per month [23]. There is no clear definition of “rich”; in official statistics, individuals who have twice the median monthly household income are usually considered as comparatively high-income earners, which was EUR 3892 per month for a single household in 2020 [24]. Hence, we assessed the monthly household net income in 4 categories: <EUR 1250, indicating that the participants live near or under the poverty line; EUR 1250–2249, implying a lower middle-class status; EUR 2250–3999, accounting for the upper-middle class; and ≥Eur 4000, meaning that participants pertain to the rich.

#### 2.3.4. Health-Related Variables

Chronic disease (yes; no; do not know) was re-classified as chronic disease awareness (yes; no (an answer of either no or do not know)); COVID-19 infection (yes, confirmed; yes, but not yet confirmed; yes, survived; no; do not know) was collapsed as disease awareness of yes (yes, confirmed; yes, but not yet confirmed; yes, survived) or no (no; do not know).

### 2.4. Statistical Analyses

Descriptive statistics with frequencies and proportions for categorical variables and unadjusted means and standard deviations (SDs) for continuous variables were calculated. We stratified the WHO-5_T_ by gender and age groups to show the fluctuation in these subgroups during the COVID-19 pandemic (at 3 time points).

For different characteristics, e.g., gender, the mean WHO-5_T_ and the percentage of participants with WHO-5_T_ ≤ 50 (defined as suspected depression) with its 95% confidence intervals were presented at each time point. To investigate whether there are subgroups that are better or worse off, we performed univariate logistic regressions of suspected depression and reported its odds ratios, including 95% confidence intervals. The potential variables were determined by univariate logistic regression with a *p*-value < 0.05 to conduct multivariate logistic regression. Thevariables included gender, age, relationship, chronic disease awareness, household size, household net income, migration background awareness, age of children and employment and status as working in the health sector. To further investigate the association between exposure variables and the risk of suspected depression, three multivariate logistic regression models with different adjustments for the covariates were performed. Model 1 was adjusted for gender, age (continuous), relationship, and chronic disease awareness, as these proved to be significant across all three observed COSMO time points. Model 2 was additionally adjusted for household size and household net income that were both significant context factors in two assessed COSMO time points. Model 3 was additionally adjusted for migration background awareness, age of children, employment, and status as working in the health sector. We also performed an overall model selection through a stepwise forward likelihood ratio test to address the leading factors. Sensitivity analysis was performed through a linear regression of the transformed WHO-5 for the predominant predictive factors, as identified by logistic model selections (Appendix E). Subgroup analyses of suspected depression were conducted for a monthly household net income stratified by the household size.

There were 2 variables with incomplete data (answering “not specified”), which were the household size (0.2–0.3%) and household net income (8–9%) (Table 1). To align with COSMO protocol, we left out missing values and only analyzed complete data. Multicollinearity between the variables was analysed with the Hosmer–Lemeshow-Ttest and factor analyses. All statistical analyses were performed using software for statistical computing in SPSS version 27.0.

## 3. Results

### 3.1. Sample Characteristics

At the three measurement points from the COSMO study, 972, 1013, and 973 different individuals in the German population were anonymously surveyed, respectively [18]. Over all three cross-sectional time points, about half of the study population was female (49.07%; 49.95%; 50.77%) and, at each time point, the overall average age lay between 44.07 to 45.92. Roughly 70% of the participants did not have children below the age of 18 years and about 4% of people having children were single parents. In time points 2 and 3, about 70% of participants were employed. Less than one-third of the people lived alone and more than two-thirds lived with others, in which half of them lived with another person and the rest lived with more than two persons. The results of the monthly household net income in time points 2 and 3 show that more than 10% live near or under the poverty line, and less than 20% are considered as wealthy. There was more than one-third of the participants who had chronic diseases among the three time points. Further results of the sample characteristics are shown in Table 1 and Table A1.

### 3.2. SWB across Different Pandemic Phases

**Figure 1:**
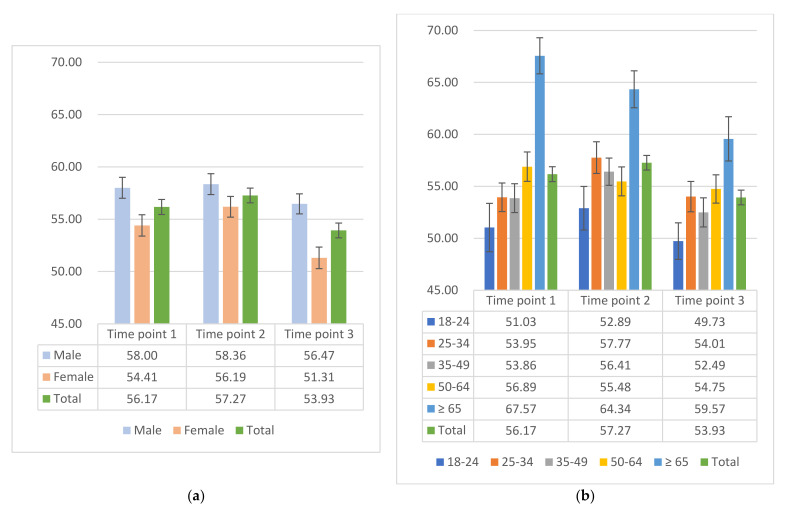
Mean WHO-5_T_ among the COSMO study at 3 time points. (**a**) Mean WHO-5_T_ of gender at different periods with a standard error and (**b**) mean WHO-5_T_ of age groups at different periods with a standard error.

The mean WHO-5_T_ scores were 56.17 (95% CI: 54.77, 57.58), 57.27 (95% CI: 55.89, 58.65), and 53.93 (95% CI: 52.54, 55.31) in the three time points, respectively. During the pandemic, the lowest average WHO-5_T_ showed in time point 3 in December 2020. According to Bech et al. [21], the score of WHO-5_T_ ranges from 0 to 100, representing the spectrum from completely absent (0) to the highest level of SWB (100), and thus indicating a mental burden in December 2020. As depicted in Figure 1, the mean WHO-5_T_ of males is higher than of females at all time points. Most notably, the difference between males and females was greater at time point 3, compared to the other time points. Considering the age groups, data showed the lowest WHO-5_T_ score for younger people (aged between 18–24 years) during the pandemic in 2020. Interestingly, the elderly population generally had the highest WHO-5_T_ during the pandemic phases. For age groups stratified by gender at each time point (Table A2), the WHO-5_T_ for females are similar for time points 1 and 2 and the lowest scores are present in time point 3 for each age group, especially for the elderly population. However, there was another pattern in the male group. The lowest WHO-5_T_ scores were not always in time point 3 for each age group.

### 3.3. Factors Associated with SWB in Different Time Points

#### 3.3.1. Univariate Analyses

- Time point 1

According to the results in Table A3, being female had a 1.46 (95% CI: 1.12, 1.90; *p*-value: 0.005)-times increased risk of suspected depression (WHO-5_T_ ≤ 50 [20]) compared to males. The risk of suspected depression among the 50–64-year age group was reduced to 55.4% (95% CI: 0.34, 0.90; *p*-value: 0.018), and the odds for suspected depression for ages above 65 years were decreased to 26.8% (95% CI: 0.15, 0.50; *p*-value < 0.001) compared to those aged between 18–24 years. Singles would have a 1.42 (*p*-value: 0.012)-times greater risk of suspected depression compared to participants in a relationship. Living alone had a significant 1.43 (*p*-value: 0.034)-times higher risk of suspected depression compared to having two persons in a household. Suffering from a chronic disease indicated a 1.60 (95% CI: 1.22, 2.10; *p*-value < 0.001)-times increased risk of suspected depression compared to no chronic disease.

- Time point 2

There was no significant difference in the risk of suspected depression between genders. Compared to the ages between 18–24 years, the risk of suspected depression was decreased to 45.5% (95% CI: 0.27, 0.76; *p*-value: 0.002) among 25–34 year-olds, and lowered to 32.4% (95% CI: 0.19, 0.56; *p*-value < 0.001) for those older than 65 years. Singles had a significant 1.50 (*p*-value: 0.003)-times increased risk of suspected depression compared to those who were in a relationship. People whose monthly net income was below EUR 1250, had a 2.00 (*p*-value < 0.001)- and 2.60 (*p*-value < 0.001)-times higher risk of suspected depression, compared to the upper-middle class (income between EUR 2250–3999) and the group with high income (income more than EUR 4000), respectively. Having a chronic disease implied a 1.92 (95% CI: 1.47, 2.52; *p*-value < 0.001)-times higher risk of suspected depression compared to no chronic disease.

- Time point 3

Similar to the results for time points 1 and 2, subjects that are female, younger, single, and suffering from chronic disease, had a higher risk of suspected depression. People with a migration background awareness had a 1.50 (95% CI: 1.09, 2.08; *p*-value: 0.013)-times higher risk of suspected depression compared to non-migration. Unemployed individuals had a 1.47 (*p*-value: 0.007)-times increased risk of suspected depression compared to employment. Living alone had a 1.54 (*p*-value: 0.011)-times increased risk of suspected depression compared to having two persons in a household. In comparison to people who lived near or under the poverty line, the more people earned indicated a reduced risk of suspected depression. The detailed proportions and odds ratios are showed in Table A3.

#### 3.3.2. Multivariate Analyses

Table A4 shows the multivariate analyses for models 1–3 at time point 1. The consistently leading variables were gender and chronic disease awareness, which indicated a 1.4 (*p*-value: 0.012)-times increased risk of suspected depression in females and nearly a 2 (*p*-value < 0.001)-times higher risk of suspected depression for people with chronic disease. The adjusted R square did not significantly improve from 0.06 in model 1 to 0.07 in model 3. Considering the multivariate results in time point 2 (Table A5), the adjusted R square improved from 0.06 in model 1 to 0.10 in model 3. The variable of household net income revealed that people with a higher income had about a 60% (*p*-value: 0.001) decrease in the risk of suspected depression compared to those living in poverty in models 2 and 3. In model 3, people who worked in the health sector had a significantly decreased risk of suspected depression by 55% (*p*-value: 0.019). With regard to time point 3 in Table 2, the consistently predictable variables were gender, age, relationship, and chronic disease awareness. The household income in models 2 and 3 showed that near poverty would have about a 2 (*p*-value < 0.05)-times higher risk of suspected depression compared to the rich group. The result of the overall model selection is provided in Table A6 and the dominant variables were not consistent across the three time points. In time point 1, gender, age, relationship, and chronic disease awareness were the influential predictors. The dominant predictors in time point 2 were age, chronic disease awareness, status as working in a health sector, and household income, and the prominent predictors in time point 3 were gender, age, chronic disease awareness, relationship, and employment.

### 3.4. Additional Analyses: Household Net Income Stratified by Household Size

The subgroup analyses for the household net income stratified by household size assessed the risk of suspected depression by WHO-5_T_ or time points 2 and 3 (data not shown). For a single household in time point 2, there was no risk difference between the various incomes; however, in time point 3, people in the lower-middle class and in the upper-middle class had a lowered suspected depression risk by 70% (*p*-value < 0.001), compared to those living below the poverty line. For a two-person household in time point 2, in comparison to households living below the poverty line, the upper-middle class and the rich group had an approximately 70% (*p*-value: 0.015, 0.002, respectively) decrease in the risk of suspected depression. We used the group with high income as a reference and the results indicate that the poverty had a 2.90 (*p*-value: 0.034)-higher risk of suspected depression in time point 3. Although the results of the subgroup analysis did not completely align across the different time points, there was still a visible result that lower income levels implied higher odds of developing depression.

## 4. Discussion

In this study, based on the COSMO data, we analyzed SWB measured using WHO-5 in repeated cross-sectional surveys within the general German adult population between the ages of 18 to 74 years. Our main result is that the SWB changes over time and the worst SWB is presented in December 2020. Possible explanations for this could be the most serious COVID-19 situation, the highest daily confirmed cases, and the strict restrictions during that time. WHO-5_T_ ≤ 50 (indicating suspected depression) was associated with being female, in the age range of 18–24 years, single, and suffering from a chronic illness in all time points.

There are various, mostly self-assessed indices to measure the well-being available. The COSMO study assessed SWB via WHO-5, which is a globally recognized and well-studied SWB instrument, which is not only validated in its German version [25], but is also recognized and commonly assessed as a tool to screen for suspected depression [20,26].

The unpredictable COVID-19 pandemic has potential burden on mental health owing to forced adaptations in lifestyles (e.g., self-isolation), travel restrictions, misinformation or disinformation, or/and the postponement of religious activities. Many of these and other factors are interrelated and may lead to lower levels of well-being during the pandemic. Based on the study results, we can observe reduced SWB compared to pre-pandemic times, which could be explained by various factors, such as mitigation and containment measures, leading to stress and adverse socioeconomic and health impacts. In our study, nearly one-third of the general population reported a poor WHO-5_T_, which increased further to about 40% during the more restrictive time in December 2020 of our observation period (Table A3). Similar results in a national survey of the general population in Italy and New Zealand reveal that almost one-third of the participants reported symptoms of anxiety or psychological distress in 2020 compared to prepandemic times [27,28]. After people underwent a sudden strict lockdown to alleviate the dissemination of the virus between mid-March and April 2020, the outbreak was under control with less than 500 newly infected cases per day. The government decided to loosen lockdown restrictions and the public still needed to follow some regulations. Our snapshot results of time point 1, from 19–20 May 2020, show lower WHO-5_T_ scores in comparison to the pre-pandemic data from a representative European study in 2016 [29]. A possible explanation is that despite the relaxation of some mitigation measures, everyday life was not the same as it was in the pre-pandemic times, and people still worried about being infected, the severity of the SARS-CoV-2 virus, the shutdown of the healthcare system or loss of loved ones, the worry of economic recession and unemployment, and finally the potential burden of home-schooling alongside having to work remotely [30,31]. With a steadying pandemic situation and a relatively minor increase in daily cases since May 2020, the daily confirmed cases were constantly increasing from August; however, the German government did not announce new rules to contain the spread of the virus. In our analysis, people felt relatively similar during time point 2, from 15–16 September 2020, compared to time point 1but still worse than during the time before the COVID-19 pandemic. In the following, intensive care units rapidly filled again in mid-October 2020 and a “lockdown light” was introduced to curb the situation. The number of infected cases amplified, and a second hard lockdown was implemented on 16 December 2020. Data from time point 3, from 21–22 December 2020, indicated a worsening of SWB. Although people already had the previous experience of dealing with the rapid spread of COVID-19 via self-protective measures, such as social distancing, wearing face masks, and hand hygiene since March 2020, the long-term pandemic circumstances indicated worse SWB and psychological burden. Restraints on physical activities (quarantine), contact with people, and visiting elderly people living in nursing homes and hopeless of the future were potentially pertinent to psychosocial distress. In this context, it is necessary to address the demand of an evolving psychological landscape.

In this study, we mainly used an indicator of suspected depression as a proxy-variable for psychological distress to interpret the study results. Sociodemographic factors, such as gender, age, ethnicity, and social roles, contribute to a certain psychological impact. Apart from gender and age, ethnic minorities tend to experience more psychological distress by virtue of stigmatization, involving lower self-esteem and access to social resources [32]. Relationship status also has an impact on psychological distress. Generally, individuals that are divorced, separated or widowed, and living alone report higher levels of psychological distress which is coherent with our findings in COSMO [33]. Our study showed that the consistent risk factor of chronic illness aligned with what was presented in existing research. People with chronic health problems tend to have adverse psychological effects and the impact seems to be long term [34,35,36]. Personal resources account for the coping strategies and studies found that a higher education and income are protective factors to limit deleterious effects on SWB for both genders, and for all age groups and across countries [34,37,38]. To date, our data did not affirm higher education as a protective factor.

The study results additionally implicate that younger people have higher odds of suspected depression in all three time points. Nearly 50% of people between 18–24 years old had lower well-being, and with an age increase, the SWB increased with it. Young adults may have age-specific challenges during COVID-19, accounting for more exposure to media, problematic Internet use, ennui due to recreation facility closures, lack of in-person contact, uncertainty of future careers, role confusion (e.g., if forced to live with parents again after independence), a change in social identification, or being poorly prepared for being an adult, such as financial self-insufficiency, owing to curtailed vocational training or entry-level employment [34,35,39]. Middle-aged adults also encountered economic and social stressors, such as increased care-giving and home-schooling for their children and concerns about their parents’ health. It was reported that the parents’ burden increased during the pandemic, but could not be replicated in our analysis [36,39]. Elderly people are a high-risk group for COVID-19 infection and its complications and vulnerable to social isolation, particularly for those living in nursing homes [30]. The WHO also emphasized the mental health of older generations and the need to provide essential support for disabled and cognitive-impaired and dementia patients [37]. With the reduction in cognitive stimulation owing to restraints from social activities, it may aggravate cognitive and behavioral symptoms of dementia [38]. Nevertheless, based on our analysis, we found relatively less psychological distress among older people, which also aligned with other studies [14,29,40]. This result may show that older people can potentially benefit from life experiences to cope with difficulties and a higher resilience compared to young people. Additionally, in our study, we only included participants up to the age of 74 years, which could not represent the overall elderly population in Germany [41]. Additionally, older people who answered this online survey may have above-average conditions and digital literacy compared to other older people.

In accordance with our study, female participants had a higher risk of suspected depression, which is consistent with the results from the WHO Global Burden of Disease Study from 2015, where the prevalence of depression was more common in women (5.1%) than in men (3.6%) [42]. These findings are also coherent with other studies conducted during the COVID-19 pandemic in that the female gender is associated with more deleterious mental health effects, such as depression and anxiety, especially in younger females [43,44]. The causes of the gender disparities are mostly unclear, however, in general, women reported more symptoms of mental health problems and perceived stress, which may be a possible explanation [13]. Psychosocial distress among females could result from a lower social status, such as cessation of work and income reduction, compared to males [45], or increased domestic violence due to physical restrictions and a stay-at-home policy [46].

Socioeconomic status (SES) is differentiated, among other things, by education level, employment status, income level, migration, and disparities in health and life expectancy. Our study found that a lower SES, a markedly lower income regarded as poverty, manifested profoundly reduced well-being during the pandemic. Unemployment also implicated higher odds of suspected depression in time point 3 in December 2020. Other studies also found that with a lower SES background, such as racial minority and joblessness, there would be a decrease in SWB and increased rates of mental disorders accounting for discrimination and disparities [27,40,47,48]. Due to the economic recession as a result of the pandemic, the loss of an existing job or potential job opportunities, the burden of daily expenses and possible extra treatment costs, might deteriorate psychosocial distress among the people in poverty, since they already live with limited resources [47]. Studies showed that the exposure of a lower socioeconomic status correlates with higher levels of distress and the distress would, in turn, affect SES [49]. Additionally, a lower SES is associated with less high-quality healthcare [50] and higher probabilities of metabolic syndromes, such as obesity, diabetes, and cardiovascular disease [51], which lead to a greater risk of severe complications and mortality once infected with COVID-19. Furthermore, they are also prone to contracting the illness due to living in a high population density, reliance on public transport, and employment in low-wage jobs, such as food and grocery services, production, and transportation, which involve intense physical contact with others and an inherent difficulty to maintain social distancing measures [52].

Within the scope of our study, we were able to identify potential groups that were particularly vulnerable during the ongoing COVID-19 pandemic, as evidenced by poorer SWB and an increased risk in suspected depression. When planning possible interventions and support measures, these groups may therefore benefit from specific consideration as well as mid-term monitoring of their mental health status.

### Strengths and Limitations

Firstly, this survey was repeated nationwide across Germany and was population-based, which represented the current status of the public perceptions and psychological impact. We acquired the potential protective and risk factors associated with one’s SWB. Most existing evidence focused on one-time cross-sectional studies and identified the short-term psychological adverse effects of COVID-19. In this study, we attempted to capture the fluctuation of COVID-19′s impact on the general society over time. Secondly, WHO-5 is a validated, widely used, and a highly practical tool that can be applied for the screening of suspected depression, and to assess SWB over time. With the high applicability of this measurement, our study could be compared to other research using this scale. As for the limitations, the cross-sectional design of snapshot data in this study was not able to assess the actual causalities between the risk factors and the mental health outcomes, but rather the observations. Secondly, COSMO data did not weight up the employment status and household size variables, which might trigger underrepresented groups. It should also be noted that the data from COSMO did not collect the information of pre-existing or previous mental illness, which might need further subgroup analyses to address the results. Thirdly, this survey was voluntary and web-based, concerning the current situation of those who participated in it. People who declined or were not willing to respond may differ from those who did in their outcomes [53].

## 5. Conclusions

This study reports lower SWB in our German adult study population during the pandemic, compared to pre-pandemic reference values. We were also able to identify some potentially protective factors as well as some risk factors associated with one’s SWB. The vulnerable groups who showed a higher risk of suspected depression were females, younger people (aged between 18–24 years), chronically ill people, people living alone, people with a migration background, or a lower socioeconomic status. Hence, these groups might particularly benefit from timely social support and psychological interventions, such as telecare systems, a 24 h available hotline consultation, and pandemic-specifically trained healthcare professionals to foster resilience resources [54,55]. It is therefore necessary to conduct further population representative longitudinal studies to inform practice and policy during the COVID-19 pandemic, in order to mitigate the negative psychological impact of the pandemic, fostering better SWB in the long term.

## Figures and Tables

**Table 1:** Sample characteristics of COSMO data in the three time points.

	Time Point 1		Time Point 2		Time Point 3	
	19–20 May 2020		15–16 September 2020		21–22 December 2020	
	n	%/Mean (SD)	n	%/Mean (SD)	n	%/Mean (SD)
Gender						
Male	477	49.07%	506	49.95%	494	50.77%
Female	495	50.93%	507	50.05%	479	49.23%
Age (cont.)	972	44.59 (15.12)	1013	45.92 (15.60)	973	44.07 (15.25)
Age group (years)						
18–24	87	8.95%	94	9.28%	118	12.13%
25–34	226	23.25%	188	18.56%	191	19.63%
35–49	266	27.37%	303	29.91%	268	27.54%
50–64	273	28.09%	276	27.25%	284	29.19%
≥65	120	12.35%	152	15.00%	112	11.51%
Education level						
No A-level	439	45.16%	478	47.19%	413	42.45%
A-level	533	54.84%	535	52.81%	560	57.55%
Migration background awareness						
No	831	85.49%	872	86.08%	789	81.09%
Yes	141	14.51%	141	13.92%	184	18.91%
Household language other than German						
No	728	74.90%	756	74.63%	725	74.51%
Yes	244	25.10%	257	25.37%	248	25.49%
Relationship status						
No	351	36.11%	374	36.92%	312	32.07%
Yes	621	63.89%	639	63.08%	661	67.93%
Age of children <18 years						
No	687	70.68%	731	72.16%	677	69.58%
0–5 years	136	13.99%	118	11.65%	152	15.62%
6–13 years	125	12.86%	159	15.70%	131	13.46%
14–17 years	81	8.33%	62	6.12%	77	7.91%
Single parent						
No	245	25.21%	237	23.40%	261	26.82%
Yes	40	4.12%	45	4.44%	35	3.60%
Employment						
No	NA	NA	350	34.55%	278	28.57%
Yes	NA	NA	663	65.45%	695	71.43%
Work in health sector						
No	876	90.12%	947	93.48%	905	93.01%
Yes	96	9.88%	66	6.52%	68	6.99%
Self-employed						
No	872	89.71%	936	92.40%	900	92.50%
Yes	100	10.29%	77	7.60%	73	7.50%
Household size						
Just me	284	29.22%	294	29.02%	228	23.43%
2 people	351	36.11%	369	36.43%	364	37.41%
≥3 people	335	34.47%	348	34.35%	378	38.85%
Not specified	2	0.21%	2	0.20%	3	0.31%
Inhabitants of hometown						
<20,000	359	36.93%	371	36.62%	379	38.95%
20,001–100,000	241	24.79%	267	26.36%	253	26.00%
100,001–500,000	183	18.83%	190	18.76%	180	18.50%
>500,000	189	19.44%	185	18.26%	161	16.55%
Household net income						
<EUR 1250	NA	NA	148	14.61%	109	11.20%
EUR 1250–2249	NA	NA	240	23.69%	238	24.46%
EUR 2250–3999	NA	NA	362	35.74%	344	35.35%
≥EUR 4000	NA	NA	182	17.97%	190	19.53%
Not specified	NA	NA	81	8.00%	92	9.46%
Chronic disease awareness						
No	625	64.30%	666	65.75%	639	65.67%
Yes	347	35.70%	347	34.25%	334	34.33%
Awareness of COVID-19 infection						
No	941	96.81%	988	97.54%	NA	NA
Yes	31	3.19%	25	2.47%	NA	NA

SD: standard deviation; NA: not available (not collected in this time point).

**Table 2:** Multivariate logistic regression of the risk of suspected depression in time point 3.

	Risk of Suspected Depression by the WHO-5_T_ in Time Point 3					
	Model 1		Model 2		Model 3	
	OR	95% CI	OR	95% CI	OR	95% CI
Gender						
Male	Ref.		Ref.		Ref.	
Female	**1.58**	[1.21, 2.05] **	**1.49**	[1.13, 1.98] **	**1.52**	[1.15, 2.03] **
Age						
Continuous	**0.98**	[0.97, 0.99] ***	**0.98**	[0.97, 0.99] **	**0.98**	[0.97, 0.99] **
Relationship status						
No	Ref.		Ref.		Ref.	
Yes	**0.56**	[0.43, 0.74] ***	**0.61**	[0.41, 0.91] *	**0.65**	[0.43, 0.97] *
Chronic disease awareness						
No	Ref.		Ref.		Ref.	
Yes	**2.12**	[1.57, 2.86] ***	**2.03**	[1.49, 2.78] ***	**2.04**	[1.49, 2.81] ***
Household size ^#^						
Just me			Ref.		Ref.	
2 persons			1.08	[0.68, 1.73]	0.99	[0.61, 1.60]
≥3 persons			1.26	[0.78, 2.04]	1.20	[0.71, 2.04]
Household income ^#^						
<EUR 1250			Ref.		Ref.	
EUR 1250–2249			**0.56**	[0.35, 0.91] *	0.63	[0.38, 1.05]
EUR 2250–3999			**0.53**	[0.32, 0.88] *	0.61	[0.36, 1.04]
≥EUR 4000			**0.42**	[0.24, 0.74] **	**0.49**	[0.26, 0.90] *
Migration background awareness						
No					Ref.	
Yes					1.36	[0.95, 1.95]
Age of children < 18years						
No					Ref.	
0–5 years					1.26	[0.81, 1.96]
6–13 years					0.63	[0.40, 1.01]
14–17 years					1.14	[0.65, 2.01]
Employment						
No					Ref.	
Yes					0.81	[0.57, 1.17]
Work in health sector						
No					Ref.	
Yes					0.78	[0.44, 1.39]
Adjusted R^2^	0.09		0.10		0.11	

**^#^** OR for logistic regression only included complete data (answer as “not specified” was excluded). * means *p* < 0.05; ** means *p* < 0.01, *** means *p* < 0.001. Bold font indicates statistical significance.

## Data Availability

https://projekte.uni-erfurt.de/cosmo2020/web/summary/ (accessed on 20 December 2021).

## Abbreviations

WHO	World Health Organization
WHO-5_T_	Transformed WHO-5
COVID-19	Coronavirus disease 2019
COSMO	COVID-19 Snapshot Monitoring
ZPID	Leibniz Institute for Psychology Information
RKI	Robert Koch Institute
BZgA	Federal Centre for Health Education
SWB	Subjective well-being
SES	Socioeconomic status
CI	Confidence interval
SD	Standard deviation
OR	Odds ratio

## Appendix A. Initial WHO-5_T_ in Different Time Points

**Table A1:** Original WHO-5_T_ in three time points.

	WHO-5_T_—Time Point 1				WHO-5_T_—Time Point 2				WHO-5_T_—Time Point 3			
	19–20 May 2020				15–16 September 2020				21–22 December 2020			
	n	%	Mean	SD	n	%	Mean	SD	n	%	Mean	SD
Total	972		56.17	22.34	1013		57.27	22.40	973		53.93	22.05
Gender												
Male	477	49.07%	58.00	21.86	506	49.95%	58.36	22.44	494	50.77%	56.47	21.27
Female	495	50.93%	54.41	22.69	507	50.05%	56.19	22.32	479	49.23%	51.31	22.54
Age group												
18–24 years	87	8.95%	51.03	21.71	94	9.28%	52.89	20.34	118	12.13%	49.73	19.11
25–34 years	226	23.25%	53.95	20.67	188	18.56%	57.77	20.91	191	19.63%	54.01	20.18
35–49 years	266	27.37%	53.86	22.64	303	29.91%	56.41	22.83	268	27.54%	52.49	22.94
50–64 years	273	28.09%	56.89	23.38	276	27.25%	55.48	23.11	284	29.19%	54.75	22.96
≥65 years	120	12.35%	67.57	19.03	152	15.00%	64.34	21.92	112	11.51%	59.57	22.54
Education level												
Up to 9 years	105	10.80%	59.50	20.02	121	11.94%	57.26	25.61	114	11.72%	50.35	26.65
At least 10 years without A-levels	334	34.36%	54.13	22.96	357	35.24%	56.40	22.91	299	30.73%	55.34	21.54
At least 10 years with A-levels	533	54.84%	56.80	22.31	535	52.81%	57.85	21.27	560	57.55%	53.90	21.23
Migration background												
Yes	141	14.51%	54.89	22.64	141	13.92%	55.49	21.80	184	18.91%	49.35	22.19
No	828	85.19%	56.36	22.28	866	85.49%	57.53	22.53	783	80.47%	54.97	21.97
Do not know	3	0.31%	64.00	32.74	6	0.59%	62.00	17.11	6	0.62%	58.00	13.57
Household language other than German												
Yes	244	25.10%	58.13	21.08	257	25.37%	57.99	22.45	248	25.49%	54.16	21.67
No	728	74.90%	55.52	22.73	756	74.63%	57.03	22.39	725	74.51%	53.85	22.19
Relationship status												
Yes	621	63.89%	57.05	22.13	639	63.08%	59.29	21.18	661	67.93%	56.21	21.59
No	351	36.11%	54.62	22.67	374	36.92%	53.83	23.98	312	32.07%	49.10	22.27
Age of children <18 years												
0–2 years	90	9.26%	54.89	23.61	77	7.60%	61.14	18.49	105	10.79%	51.96	21.77
3–5 years	64	6.58%	49.25	22.28	55	5.43%	55.78	21.05	62	6.37%	55.74	21.37
6–9 years	77	7.92%	53.61	23.19	87	8.59%	56.05	20.51	77	7.91%	62.29	18.54
10–13 years	72	7.41%	49.61	23.91	107	10.56%	56.71	23.79	74	7.61%	53.03	22.39
14–17 years	81	8.33%	61.63	20.64	62	6.12%	55.42	22.06	77	7.91%	51.48	23.58
No	687	70.68%	56.79	21.86	731	72.16%	57.54	22.66	677	69.58%	54.24	22.29
Single parent												
Yes	40	4.12%	53.90	22.59	45	4.44%	55.02	22.87	35	3.60%	48.69	23.45
No	245	25.21%	54.82	23.61	237	23.40%	56.88	21.53	261	26.82%	53.82	21.20
Employment												
Yes	NA	NA	NA	NA	663	65.45%	57.24	21.72	695	71.43%	55.21	21.00
No	NA	NA	NA	NA	350	34.55%	57.34	23.66	278	28.57%	50.73	24.22
Work in health sector												
Yes	96	9.88%	53.17	21.63	66	6.52%	60.61	21.38	68	6.99%	54.18	21.73
No	876	90.12%	56.50	22.41	947	93.48%	57.04	22.46	905	93.01%	53.91	22.08
Self-employed												
Yes	100	10.29%	57.04	22.51	77	7.60%	58.55	20.96	73	7.50%	54.85	22.59
No	872	89.71%	56.07	22.34	936	92.40%	57.17	22.52	900	92.50%	53.85	22.02
Household size												
Just me	284	29.22%	55.63	22.62	294	29.02%	55.16	24.17	228	23.43%	51.40	23.38
2 persons	351	36.11%	57.89	21.87	369	36.43%	59.36	21.14	364	37.41%	56.37	21.77
3–4 persons	276	28.40%	55.67	23.04	297	29.32%	57.41	21.54	323	33.20%	52.15	20.88
More than 4 persons	59	6.07%	51.73	19.75	51	5.03%	53.25	24.52	55	5.65%	58.98	23.37
Not specified	2	0.21%	32.00	22.63	2	0.20%	64.00	28.28	3	0.31%	48.00	14.42
Inhabitants of hometown												
≤5000	148	15.23%	57.57	23.79	168	16.58%	59.74	20.50	136	13.98%	52.91	22.69
5001–20,000	211	21.71%	57.50	20.27	203	20.04%	55.53	22.54	243	24.97%	52.87	22.18
20,001–100,000	241	24.79%	56.73	21.95	267	26.36%	58.65	22.12	253	26.00%	53.75	21.95
100,001–500,000	183	18.83%	53.42	23.16	190	18.76%	55.14	23.03	180	18.50%	54.42	20.71
>500,000	189	19.44%	55.56	23.05	185	18.26%	57.15	23.47	161	16.55%	56.10	23.00
Household income												
<EUR 1250	NA	NA	NA	NA	148	14.61%	49.27	24.82	109	11.20%	44.07	25.65
EUR 1250–1749	NA	NA	NA	NA	117	11.55%	56.44	22.84	115	11.82%	50.61	21.85
EUR 1750–2249	NA	NA	NA	NA	123	12.14%	55.38	23.19	123	12.64%	56.78	21.83
EUR 2250–2999	NA	NA	NA	NA	169	16.68%	56.45	22.36	181	18.60%	55.05	21.57
EUR 3000–3999	NA	NA	NA	NA	193	19.05%	61.08	20.01	163	16.75%	55.95	19.92
EUR 4000–4999	NA	NA	NA	NA	105	10.37%	59.01	20.60	122	12.54%	56.66	19.74
≥EUR 5000	NA	NA	NA	NA	77	7.60%	66.23	18.83	68	6.99%	59.18	20.47
Not specified	NA	NA	NA	NA	81	8.00%	57.83	22.25	92	9.46%	52.65	23.21
Chronic disease												
Yes	347	35.70%	51.87	23.03	347	34.25%	51.09	24.29	334	34.33%	49.07	23.23
No	600	61.73%	58.93	21.46	643	63.47%	60.89	20.54	608	62.49%	56.82	20.90
Do not know	25	2.57%	49.76	23.78	23	2.27%	49.39	20.77	31	3.19%	49.55	21.71
COVID-19 infection												
Yes, confirmed	11	1.13%	59.27	21.53	14	1.38%	62.29	24.09	NA	NA	NA	NA
Yes, but not yet confirmed	9	0.93%	49.78	14.44	5	0.49%	41.60	24.27	NA	NA	NA	NA
No	803	82.61%	56.86	22.73	923	91.12%	57.84	22.38	NA	NA	NA	NA
Yes, survived	11	1.13%	50.18	17.01	6	0.59%	48.00	25.17	NA	NA	NA	NA
Do not know	138	14.20%	52.84	20.65	65	6.42%	50.22	20.58	NA	NA	NA	NA

NA: not available (not collected in this time point).

## Appendix B. WHO-5_T_ Stratified by Gender and Age Group

**Table A2:** WHO-5_T_ stratified by gender and age group in three time points.

	WHO-5_T_ in Time Point 1		WHO-5_T_ in Time Point 2		WHO-5_T_ in Time Point 3	
	19–20 May 2020		15–16 September 2020		21–22 December 2020	
	Partial Lockdown		No Restrictions		2nd Strict Lockdown	
	Mean	SD	Mean	SD	Mean	SD
Female (Total)	54.41 ^2^	22.69	56.19 ^3^	22.32	51.31 ^2,3^	22.54
18–24	45.62 ^1^	22.94	54.26 ^1^	19.02	46.84	18.95
25–34	53.88	21.67	55.18	20.54	52.88	21.14
35–49	52.58	22.25	53.95	22.43	49.58	22.94
50–64	55.94	23.72	53.71	23.82	51.62	23.80
≥65	63.84	20.36	66.90 ^3^	20.61	57.33 ^3^	23.48
Men (Total)	58.00	21.86	58.36	22.44	56.47	21.27
18–24	56.09	19.42	51.58	21.65	52.25	19.05
25–34	54.04	19.39	60.53	21.06	55.25	19.11
35–49	55.25	23.06	58.70	23.03	55.32	22.66
50–64	57.84	23.09	57.25	22.32	58.10	21.61
≥65	70.32 ^1,2^	17.63	61.50 ^1^	23.10	61.07 ^2^	21.94
Overall	56.17 ^2^	22.34	57.27 ^3^	22.40	53.93 ^2,3^	22.05

^1^: WHO-5_T_ is significantly different between time points 1 and 2; ^2^: WHO-5_T_ is significantly different between time points 1 and 3; and ^3^: WHO-5_T_ is significantly different between time points 2 and 3. Significant difference is defined as *p*-value < 0.05.

## Appendix C. Univariate Analyses of Risk of Suspected Depression by the WHO-5_T_ in Three Time Points

**Table A3:** Univariate logistic regression of rick of suspected depression in three time points.

Risk of Suspected Depression by the WHO-5_T_ in COSMO												
	Time Point 1				Time Point 2				Time Point 3			
	19–20 May 2020				15–16 September 2020				21–22 December 2020			
	% of Depression ^1^	95% CI	OR	95% CI	% of Depression	95% CI	OR	95% CI	% of Depression	95% CI	OR	95% CI
Gender												
Male (reference)	31.24%	[29.14, 33.34]			32.02%	[27.90, 36.13]			35.22%	[31.01–39.44]		
Female	39.80%	[37.60, 42.00]	1.46	[1.12, 1.90] **	37.48%	[33.16, 41.79]	1.27	[0.98, 1.65]	46.56%	[42.08–51.03]	1.60	[1.24, 2.07] ***
Age (continuous)	35.60%	[32.59, 38.61]	0.99	[0.98, 0.99] **	34.75%	[31.81, 37.68]	0.99	[0.98, 1.00] *	40.80%	[37.71–43.89]	0.99	[0.98, 0.99] **
Age group (years)												
18–24 (reference)	48.28%	[42.89, 53.66]			48.94%	[38.78, 59.10]			53.39%	[44.35–62.43]		
25–34	37.17%	[33.95, 40.39]	0.63	[0.39, 1.05]	30.32%	[23.73, 36.91]	0.45	[0.27, 0.76] **	41.88%	[34.87–48.90]	0.63	[0.40, 1.00] *
35–49	38.72%	[35.73, 41.71]	0.68	[0.42, 1.10]	35.97%	[30.56, 41.39]	0.59	[0.37, 0.94] *	41.04%	[35.14–46.95]	0.61	[0.39, 0.94] *
50–64	34.07%	[31.19, 36.94]	0.55	[0.34, 0.90] *	37.68%	[31.95, 43.41]	0.63	[0.39, 1.01]	38.73%	[33.06–44.41]	0.55	[0.39, 0.85] **
≥65	20.00%	[12.81, 27.19]	0.27	[0.15, 0.50] ***	23.68%	[16.90, 30.47]	0.32	[0.19, 0.56] ***	30.36%	[21.80–38.91]	0.38	[0.22, 0.65] ***
Education level												
No A-level (reference)	37.59%	[33.05, 42.12]			36.19%	[31.88, 40.51]			38.98%	[34.27–43.69]		
A-level	33.96%	[29.93, 37.98]	0.85	[0.66, 1.11]	33.46%	[29.46, 37.46]	0.89	[0.68, 1.15]	42.14%	[38.05–46.24]	1.14	[0.88, 1.48]
Migration background awareness												
No (reference)	34.78%	[31.54, 38.02]			34.40%	[31.25, 37.56]			38.91%	[35.51–42.31]		
Yes	40.43%	[32.30, 48.55]	1.27	[0.88, 1.83]	36.88%	[28.89, 22.87]	1.11	[0.77, 1.61]	48.91%	[41.67–56.16]	1.50	[1.09, 2.08] *
Household language other than German												
No (reference)	36.95%	[33.44, 40.46]			35.45%	[32.04, 38.86]			40.69%	[37.11–44.27]		
Yes	31.56%	[25.71, 37.40]	0.79	[0.58, 1.07]	32.68%	[26.94, 38.43]	0.88	[0.66, 1.19]	41.13%	[34.99–47.27]	1.02	[0.76, 1.37]
Relationship status												
No (reference)	40.74%	[35.59, 45.89]			40.64%	[35.66, 45.63]			51.60%	[46.05–57.16]		
Yes	32.69%	[29.00, 36.38]	0.71	[0.54, 0.93] *	31.30%	[27.70, 34.90]	0.67	[0.51, 0.87] **	35.70%	[32.05–39.36]	0.52	[0.40, 0.68] ***
Age of children <18 years												
No (reference)	35.08%	[31.51, 38.65]			35.29%	[31.83, 38.76]			40.77%	[37.06–44.47]		
0–5 years	38.24%	[30.04, 46.43]	1.08	[0.74, 1.57]	27.12%	[19.06, 35.17]	0.66	[0.43, 1.02]	43.42%	[35.52–51.33]	1.21	[0.85, 1.73]
6–13 years	42.40%	[33.70, 51.10]	1.45	[0.98, 2.14]	36.48%	[28.97, 43.98]	1.10	[0.77, 1.58]	33.59%	[25.47–41.71]	0.66	[0.45, 0.99] *
14–17 years	27.16%	[17.41, 36.91]	0.62	[0.37, 1.03]	40.32%	[28.01, 52.63]	1.25	[0.73, 2.13]	42.86%	[31.73–53.98]	1.21	[0.75, 1.95]
Single parent												
No (reference)	36.73%	[30.69, 42.78]			33.76%	[27.72, 39.79]			40.61%	[34.64–46.58]		
Yes	37.50%	[22.31, 52.69]	1.03	[0.52, 2.06]	31.11%	[17.43, 44.79]	0.89	[0.45, 1.76]	42.86%	[26.22–59.49]	1.10	[0.54, 2.24]
Employment												
No (reference)	NA	NA			34.86%	[29.86, 39.86]			47.48%	[41.60–53.36]		
Yes	NA	NA	NA	NA	34.69%	[31.06, 38.32]	0.99	[0.76, 1.30]	38.13%	[34.52–41.74]	0.68	[0.52, 0.90] **
Work in health sector												
No (reference)	35.27%	[32.11, 38.44]			35.48%	[32.43, 38.53]			41.10%	[37.90–44.31]		
Yes	38.54%	[28.75, 48.33]	1.15	[0.75, 1.78]	24.24%	[13.82, 34.66]	0.58	[0.33, 1.04]	36.76%	[25.22–48.31]	0.83	[0.50, 1.39]
Self-employed												
No (reference)	35.21%	[32.03, 38.38]			34.83%	[31.78, 37.88]			41.00%	[37.78–44.22]		
Yes	39.00%	[29.39, 48.61]	1.18	[0.77, 1.80]	33.77%	[23.13, 44.40]	0.95	[0.58, 1.56]	38.36%	[27.12–49.59]	0.90	[0.55, 1.46]
Household size ^#^												
Just me (reference)	38.73%	[33.06, 44.41]			37.41%	[31.87, 42.96]			46.49%	[40.00–52.98]		
2 people	30.77%	[25.93, 35.60]	0.70	[0.51, 0.98] *	31.98%	[27.21, 36.74]	0.79	[0.57, 1.09]	35.99%	[31.05–40.93]	0.65	[0.46, 0.91] *
≥ 3 people	37.61%	[32.42, 42.81]	0.95	[0.69, 1.32]	35.34%	[30.31, 40.37]	0.91	[0.66, 1.26]	42.06%	[37.08–47.05]	0.87	[0.60, 1.16]
Inhabitants of hometown												
≤20,000 (reference)	32.59%	[27.74, 37.45]			35.31%	[30.44, 40.18]			43.54%	[38.54–48.53]		
20,001–100,000	36.51%	[30.42, 42.61]	1.19	[0.84, 1.68]	32.21%	[26.59, 37.83]	0.87	[0.62, 1.22]	40.32%	[34.26–46.37]	0.88	[0.63, 1.21]
100,001–500,000	37.16%	[30.14, 44.18]	1.22	[0.84, 1.78]	36.32%	[29.46, 43.17]	1.05	[0.73, 1.50]	38.89%	[31.75–46.03]	0.83	[0.58, 1.19]
>500,000	38.62%	[31.66, 45.58]	1.30	[0.90, 1.88]	35.68%	[28.75, 42.60]	1.02	[0.70, 1.47]	37.27%	[29.77–44.76]	0.77	[0.53, 1.13]
Household income ^#^												
<EUR 1250 (reference)	NA	NA			48.65%	[40.57, 56.73]			60.55%	[51.33–69.77]		
EUR 1250–2249	NA	NA	NA	NA	38.75%	[32.57, 44.93]	0.67	[0.44, 1.01]	43.28%	[36.97–49.59]	0.50	[0.31, 0.79] **
EUR 2250–3999	NA	NA	NA	NA	30.39%	[25.64, 35.13]	0.46	[0.31, 0.68] ***	37.21%	[32.09–42.32]	0.39	[0.25, 0.60] ***
≥EUR 4000	NA	NA	NA	NA	24.73%	[18.44, 31.01]	0.35	[0.22, 0.55] ***	32.63%	[25.95–39.32]	0.32	[0.19, 0.52] ***
Chronic disease awareness												
No (reference)	31.68%	[28.03, 35.33]			29.58%	[26.11, 33.05]			36.46%	[32.73–40.20]		
Yes	42.65%	[37.44, 47.86]	1.60	[1.22, 2.10] **	44.67%	[39.43, 49.91]	1.92	[1.47, 2.52] ***	49.10%	[43.73–54.47]	1.68	[1.29, 2.20] ***
Awareness of COVID-19 infection												
No (reference)	35.49%	[32.44, 38.55]			34.72%	[31.75, 37.69]			NA	NA		
Yes	38.71%	[21.28, 56.14]	1.15	[0.55, 2.39]	36.00%	[16.80, 55.20]	1.06	[0.46, 2.42]	NA	NA	NA	NA

^1^ Elevated rick of suspected depression represented by the WHO-5_T_ ≤ 50; ^#^ OR for logistic regression only included complete data (answer as “not specified” was excluded). * means *p* < 0.05; ** means *p* < 0.01, *** means *p* < 0.001. Bold font indicates statistical significance. NA: not available (not collected in this time point).

## Appendix D. Multivariate Analyses of Risk of Suspected Depression by the WHO-5_T_

**Table A4:** Multivariate logistic regression for the risk of suspected depression in time point 1.

	Risk of Suspected Depression by the WHO-5_T_ in Time Point 1					
	Model 1		Model 2		Model 3	
	OR	95% CI	OR	95% CI	OR	95% CI
Gender						
Male	Ref.		Ref.		Ref.	
Female	**1.41**	**[1.08, 1.85] ****	**1.39**	**[1.06, 1.83] ***	**1.40**	**[1.06, 1.84] ***
Age						
Continuous	**0.98**	**[0.97, 0.99] *****	**0.98**	**[0.97, 0.99] *****	**0.98**	**[0.97, 0.99] ****
Relationship status						
No	Ref.		Ref.		Ref.	
Yes	**0.75**	**[0.57, 0.99] ***	0.77	[0.53, 1.12]	0.77	[0.52, 1.13]
Chronic disease awareness						
No	Ref.		Ref.		Ref.	
Yes	**1.87**	**[1.39, 2.50] *****	**1.88**	**[1.40, 2.53] *****	**1.88**	**[1.40, 2.54] *****
Household size ^#^						
Just me			Ref.		Ref.	
2 persons			0.90	[0.58, 1.38]	0.89	[0.57, 1.38]
≥3 persons			1.07	[0.67, 1.65]	1.05	[0.65, 1.69]
Migration background awareness						
No					Ref.	
Yes					1.14	[0.78, 1.68]
Age of children <18 years						
No					Ref.	
0–5 years					1.03	[0.66, 1.61]
6–13 years					1.38	[0.89, 2.14]
14–17 years					0.67	[0.38, 1.18]
Work in health sector						
No					Ref.	
Yes					0.94	[0.59, 1.48]
Adjusted R^2^	0.06		0.06		0.07	

**^#^** OR for logistic regression only included complete data (answer as “not specified” was excluded). * means *p* < 0.05; ** means *p* < 0.01, *** means *p* < 0.001. Bold font indicates statistical significance.

**Table A5:** Multivariate logistic regression for the risk of suspected depression in time point 2.

	Risk of Suspected Depression by the WHO-5_T_ in Time Point 2					
	Model 1		Model 2		Model 3	
	OR	95% CI	OR	95% CI	OR	95% CI
Gender						
Male	Ref.		Ref.		Ref.	
Female	1.29	[0.99, 1.69]	1.29	[0.97, 1.71]	**1.34**	**[1.01, 1.78] ***
Age						
Continuous	**0.98**	**[0.98, 0.99] ****	**0.99**	**[0.98, 1.00] ***	**0.99**	**[0.98, 1.00] ***
Relationship status						
No	Ref.		Ref.		Ref.	
Yes	**0.70**	**[0.54, 0.92] ***	0.82	[0.55, 1.21]	0.85	[0.57, 1.28]
Chronic disease awareness						
No	Ref.		Ref.		Ref.	
Yes	**2.20**	**[1.65, 2.92] *****	**2.04**	**[1.51, 2.76] *****	**2.14**	**[1.57, 2.91] *****
Household size ^#^						
Just me			Ref.		Ref.	
2 persons			1.17	[0.75, 1.82]	1.19	[0.76, 1.88]
≥3 persons			1.34	[0.84, 2.14]	1.34	[0.80, 2.24]
Household income ^#^						
<EUR 1250			Ref.		Ref.	
EUR 1250–2249			0.73	[0.47, 1.13]	0.68	[0.43, 1.08]
EUR 2250–3999			**0.46**	**[0.29, 0.72] ****	**0.41**	**[0.25, 0.67] *****
≥EUR 4000			**0.38**	**[0.22, 0.66] ****	**0.34**	**[0.19, 0.60] *****
Migration background awareness						
No					Ref.	
Yes					1.11	[0.74, 1.67]
Age of children <18 years						
No					Ref.	
0–5 years					0.66	[0.40, 1.08]
6–13 years					1.16	[0.75, 1.79]
14–17 years					1.21	[0.65, 2.25]
Employment						
No					Ref.	
Yes					1.29	[0.90, 1.85]
Work in health sector						
No					Ref.	
Yes					**0.45**	**[0.23, 0.88] ***
Adjusted R^2^	0.06		0.08		0.10	

**^#^** OR for logistic regression only included complete data (answer as “not specified” was excluded). * means *p* < 0.05; ** means *p* < 0.01, *** means *p* < 0.001. Bold font indicates statistical significance.

**Table A6:** Model selection of risk of suspected depression in three time points.

	Risk of Suspected Depression by the WHO-5_T_		
	Time Point 1	Time Point 2	Time Point 3
	19–20 May 2020	15–16 September 2020	21–22 December 2020
	OR 95% CI	OR 95% CI	OR 95% CI
Gender			
Male	Ref.		Ref.
Female	**1.40 [1.07, 1.83] ***		**1.52 [1.15, 2.01] ****
Age (continuous)	**0.98 [0.97, 0.99] *****	**0.99 [0.98, 1.00] ****	**0.98 [0.97, 0.99] *****
Relationship status			
No	Ref.		Ref.
Yes	**0.75 [0.57, 0.99] ***		**0.56 [0.41, 0.75] *****
Chronic disease awareness			
No	Ref.	Ref.	Ref.
Yes	**1.87 [1.39, 2.50] *****	**2.08 [1.54, 2.82] *****	**2.00 [1.46, 2.73] *****
Employment			
No			Ref.
Yes			**0.68 [0.49, 0.95] ***
Work in health sector			
No		Ref.	
Yes		**0.49 [0.26, 0.94] ***	
Household income			
<EUR 1250		Ref.	
EUR 1250–2249		0.77 [0.50, 1.17]	
EUR 2250–3999		**0.49 [0.33, 0.73] *****	
≥EUR 4000		**0.40 [0.25, 0.64] *****	
Adjusted R^2^	0.06	0.08	0.09

* means *p* < 0.05; ** means *p* < 0.01, *** means *p* < 0.001. Bold font indicates statistical significance.

## Appendix E. Sensitivity Analysis

As mentioned above, dominant predictors are not completely consistent across the time points; therefore, we adopted relative predictive factors from model selections in Table A6 and performed sensitivity analysis via linear regression of the WHO-5_T_ to test the main predictive variables at different time points. The result is fairly consistent with the significant results in Table A7.

**Table A7:** Multivariate linear regression of the WHO-5_T_ in three time points.

	Linear Regression of the WHO-5_T_ in COSMO					
	Time Point 1		Time Point 2		Time Point 3	
	19–20 May 2020		15–16 September 2020		21–22 December 2020	
	Beta	95% CI	Beta	95% CI	Beta	95% CI
Gender						
Male						
Female	**−2.80**	**[−5.54, −0.07] ***			**−4.53**	**[−7.19, −1.87] ****
Age	**0.30**	**[0.21, 0.40] *****	**0.22**	**[0.13, 0.32] *****	**0.27**	**[0.17, 0.36] *****
Relationship status						
No						
Yes	1.24	[−1.62, 4.10]			**5.32**	**[2.43, 8.21] *****
Chronic disease awareness						
No						
Yes	**−9.14**	**[−12.12, −6.15] *****	**−10.00**	**[−13.04, −6.97] *****	**−9.27**	**[−12.26, −6.28] *****
Employment						
No						
Yes					**4.42**	**[1.31, 7.53] ****
Work in health sector						
No						
Yes			4.66	[−1.15, 10.48]		
Household income						
<EUR 1250						
EUR 1250–2249			**4.55**	**[0.09, 9.00] ***		
EUR 2250–3999			**8.41**	**[4.27, 12.55] *****		
≥EUR 4000			**10.64**	**[5.91, 15.37] *****		

* means *p* < 0.05; ** means *p* < 0.01, *** means *p* < 0.001. Bold font indicates statistical significance.

## Appendix F. Measured Variables: Question and Answer Categories

**Table A8:** Question and answer categories.

Variable	Question	Answer Categories
Gender	What is your gender?	1—Male 2—Female
Age	How old are you?	I am___ years old Recoded to: AGE_Level 1—18 to 24 years 2—25 to 34 years 3—35 to 49 years 4—50 to 64 years 5—≥65 years
Education	How many years of education have you completed?	1—0–9 years 2—at least 10 years without A-Level 3—at least 10 years with A-Level Recoded to: Edu_With_A_level 0—no A-Level 1—A-Level
Migration	Are you aware of yourself or any of your parents being born abroad?	1—Yes 2—No 99—Do not know Recoded to: Mig_Aware_Y 0—No or Do not know 1—Yes
House_Language	Do you mainly speak a language other than German in your household?	1—Yes 2—No Recoded to: Langu_Other_G 0—No 1—Yes
IPV_Partner_1	Are you in a relationship or partnership (including marriage)?	1—Yes 2—No Recoded to: Partner_Y 0—No 1—Yes
Child_0_B3 Child_3_B6 Child_6_B10 Child_10_B14 Child_14_B18 Child_NO	Do you have one or more children under the age of 18?	CHILD_0_B3 0 to under 3 years CHILD_3_B6 3 to under 6 years CHILD_6_B10 6 to under 10 years CHILD_10_B14 10 to under 14 years CHILD_14_B18 14 to under 18 years CHILD_NO no children under 18 years 0—Not quoted 1—Quoted Recoded to: child_0_6 (0 to under 6 years) child_6_14 (6 to under 14 years) 0—Not quoted 1—Quoted
Single_Parent	Are you a single parent?	1—Yes 2—No Recoded to: Sin_Pare_Y 0—No 1—Yes
Employment	Are you employed?	1—Yes 2—No Recoded to: Employ_Y 0—No 1—Yes
Health	Do you have a job in the health sector?	1—Yes 2—No Recoded to: Healthjob_Y 0—No 1—Yes
Self_Employed	Are you a freelancer or self-employed?	1—Yes 2—No Recoded to: Self_employ_Y 0—No 1—Yes
Household_Size	How many people permanently live in your household?	1—Just me 2—2 persons 3—3–4 persons 4—More than 4 persons 88—Not specified Recoded to: Housesize_C_123 1—Just me 2—2 persons 3—≥3 persons 88—Not specified
Inhabitants	How many inhabitants live in the village or town in which you live?	1—≤5000 2—5001–20,000 3—20,001–100,000 4—100,001–500,000 5—>500,000 Recoded to: Inhab_C 1—≤20,000 2—20,001–100,000 3—100,001–500,000 4—>500,000
Income_HH	What is the total monthly net income of your household?	1—<EUR 1250 2—EUR 1250–1749 3—EUR 1750–2249 4—EUR 2250–2999 5—EUR 3000–3999 6—EUR 4000–4999 7—≥EUR 5000 88—Not specified Recoded to: Income_C 0—<EUR 1250 1—EUR 1250–2249 2—EUR 2250–3999 3—≥EUR 4000 88—Not specified
Chronic	Do you have a chronic disease?	1—Yes 2—No 99—Do not know Recoded to: Chro_Aware_Y 0—No or Do not know 1—Yes
Infected	Have you or were you infected with the novel coronavirus?	1—Yes, confirmed 2—Yes, but not yet confirmed 11—Yes, survived 3—No 99—Do not know Recoded to: Infect_Aware_Y 0—No or Do not know 1—Yes (confirmed, not yet confirmed, or survived)
Instruction for WHO-5 index questionnaire:	The following statements relate to your well-being in the past two weeks. For each statement, please mark the rubric that you think best describes how you have felt in the past two weeks.	
WHO5_1	I have felt cheerful and in good spirits.	1—All of the time 2—Most of the time 3—More than half the time 4—Less than half the time 5—Some of the time 6—At no time Recoded to: WHO5_R_1 0—At no time 1—Some of the time 2—Less than half the time 3—More than half the time 4—Most of the time 5—All of the time
WHO5_2	I have felt calm and relaxed.	1—All of the time 2—Most of the time 3—More than half the time 4—Less than half the time 5—Some of the time 6—At no time Recoded to: WHO5_R_2 0—At no time 1—Some of the time 2—Less than half the time 3—More than half the time 4—Most of the time 5—All of the time
WHO5_3	I have felt active and vigorous.	1—All of the time 2—Most of the time 3—More than half the time 4—Less than half the time 5—Some of the time 6—At no time Recoded to: WHO5_R_3 0—At no time 1—Some of the time 2—Less than half the time 3—More than half the time 4—Most of the time 5—All of the time
WHO5_4	I woke up feeling fresh and rested.	1—All of the time 2—Most of the time 3—More than half the time 4—Less than half the time 5—Some of the time 6—At no time Recoded to: WHO5_R_4 0—At no time 1—Some of the time 2—Less than half the time 3—More than half the time 4—Most of the time 5—All of the time
WHO5_5	My daily life has been filled with things that interest me.	1—All of the time 2—Most of the time 3—More than half the time 4—Less than half the time 5—Some of the time 6—At no time Recoded to: WHO5_R_5 0—At no time 1—Some of the time 2—Less than half the time 3—More than half the time 4—Most of the time 5—All of the time
		Sum of WHO5_R_1 to WHO5_R_5 Recoded to: WHO5_R_sum WHO5_R_sum multiplied by 4 Recoded to: WHO5_T_sum WHO5_T_sum Recoded to: WHO_depression 0—WHO5_T_sum > 50 1—WHO5_T_sum ≤ 50
